# CIHR Health System Impact Fellows: Reflections on "Driving Change" Within the Health System

**DOI:** 10.15171/ijhpm.2018.124

**Published:** 2018-12-30

**Authors:** S Meaghan Sim, Jonathan Lai, Katie Aubrecht, Ivy Cheng, Mark Embrett, El Kebir Ghandour, Megan Highet, Rebecca Liu, Christiane PM Casteli, Margaret Saari, Samiratou Ouédraogo, Hazel Williams-Roberts

**Affiliations:** ^1^ Healthy Populations Institute, Dalhousie University, Halifax, NS, Canada.; ^2^ Nova Scotia Health Authority, Halifax, NS, Canada.; ^3^ McGill University, Montreal, QC, Canada.; ^4^ Centre for Innovation in Autism and Intellectual Disabilities, Montreal, QC, Canada.; ^5^ Mount Saint Vincent University, Halifax, NS, Canada.; ^6^ Continuing-Care Research, Nova Scotia Health Authority, Halifax, NS, Canada.; ^7^ Institute of Health Policy and Management, University of Toronto, Toronto, ON, Canada.; ^8^ Sunnybrook Health Sciences Centre, Toronto, ON, Canada.; ^9^ DeGroote School of Business, McMaster University, Hamilton, ON, Canada.; ^10^ Nova Scotia Health Research Foundation, Halifax, NS, Canada.; ^11^ Institut National D’excellence en Santé et en Services Sociaux (INESSS), Québec City, QC, Canada.; ^12^ Centre Intégré en Santé et Services Sociaux de Chaudière-Appalaches, Ste-Marie, QC, Canada.; ^13^ Département de Médecine Familiale et Médecine D’urgence, Université Laval, Quebec City, QC, Canada.; ^14^ School of Public Health, University of Alberta, Edmonton, AB, Canada.; ^15^ Primary and Community Health, Health Service Delivery, Alberta Health, Edmonton, AB, Canada.; ^16^ Department of Human Kinetics, University of Ottawa, Ottawa, ON, Canada.; ^17^ University Health and Social Services Centre (IUHSSC) of Capitale-Nationale (CN), Faculty of Nursing Sciences, Université Laval, Québec City, QC, Canada.; ^18^ School of Public Health and Health Systems, University of Waterloo, Waterloo, ON, Canada.; ^19^ SE Health, Markham, ON, Canada.; ^20^ Department of Epidemiology Epidemiology, Biostatistics and Occupational Health, McGill University, Montréal, QC, Canada.; ^21^ Institut National de Santé Publique du Québec (INSPQ), Montréal, QC, Canada.; ^22^ Saskatchewan Health Authority, Saskatoon, SK, Canada.; ^23^ University of Saskatchewan, Saskatoon, SK, Canada.

**Keywords:** Embedded Researcher, Postdoctoral Training, Learning Health Systems, Integrated Knowledge Translation (IKT), Canada

## Abstract

Learning health systems necessitate interdependence between health and academic sectors and are critical to address the present and future needs of our health systems. This concept is being supported through the new Canadian Institutes of Health Research (CIHR) Health System Impact (HSI) Fellowship, through which postdoctoral fellows are situated within a health system-related organization to help propel evidence-informed organizational transformation and change. A voluntary working group of fellows from the inaugural cohort representing diversity in geography, host setting and personal background, collectively organized a panel at the 2018 Canadian Association for Health Services and Policy Research Conference with the purpose of describing this shared scholarship experience. Here, we present a summary of this panel reflecting on our experiential learning in a practice environment and its ability for impact.

## Background

The complexity of today’s health systems requires integration and interdependence among health and academic sectors.^1,2^ The “know-do” gap that continues to exist between research and practice presents an ongoing challenge for substantive health system transformation, and is a lost opportunity for both the health system and society as a whole.^3^ As proportionately fewer PhD graduates follow the traditional academic career path,^4^ there is also a need to ensure that these individuals are ready to respond to present and future health system needs.^5^ Such are foundational concepts behind Canadian Institutes of Health Research’s (CIHR’s) new Health System Impact (HSI) Fellowship launched in 2017-2018, through which postdoctoral fellows (n = 46) were co-located between a health system-related organization (50%-100% full-time equivalent [FTE]) and an academic institution. As one of the key initiatives of the Canadian Health Services and Policy Research Alliance’s Training Modernization Strategy, and the first pan-Canadian initiative of its kind, the fellowship is designed to provide a high quality, postdoctoral training environment for fellows to learn about and address critical health system challenges; encourage professional development within an expanded set of competencies; and to optimize the use of evidence within health system organizations to support functioning as learning health systems.^4,5^ The outcomes of embedding early career researchers in this manner have yet to be fully realized.

Understanding the processes of integrated knowledge translation (IKT) interventions is a challenge owing to the complexity of studying multifaceted social interactions and resultant change within heterogeneous contexts^6^ and inconsistencies in the way IKT is applied.^7^ Embedding researchers within health system organizations is recognized as an enabler for IKT to occur,^7^ through strengthening the relationship between researchers and decision-makers for engaging in co-produced research that impacts decisions made by the organization.^8^ There is increasing global precedent for embedding researchers as an approach to support quality improvement and strengthen health systems.^8-10^

In supporting KT, the fellowship aims to bridge the knowledge-to-practice gap though propelling evidence-informed improvements in health services and health policy. This goal is primarily supported through the HSI fellows’ work on identified impact projects within their host organizations; several authors described their projects as IKT in nature. Beyond a broad goal of HSI,^11^ and contributions to culture change in both academia and health systems,^12^ this unique training opportunity also enables personal transformation. HSI fellows are trained to step into emerging roles as future health system and policy leaders within a complex and evolving landscape. As a subset of the inaugural cohort of HSI Fellows^13^ we reflect on our learning journey to consider how this fellowship has influenced the contributions made in support of HSI.^11^

### A Framework for the HSI Fellow as an Embedded Researcher

Although anecdotal reports from our working group indicated a heterogeneous fellowship experience, we reflected upon framing the fellowship through a common lens to bring cohesion to the disparate experiences, both personally and within the organizations in which we were embedded. A proposed framework for the embedded HSI fellow, created by JL, was valuable for this purpose (Figure). This framework was inspired by the work of Boyer^14^ and Mentzer,^15^ that is relevant to both scholarly and practice-based environments. Coupled with reflections about our early lessons as HSI fellows (both shared and distinctive), the figure depicts the HSI fellow as a central agent, with inputs and feedback loops representing the processes, outputs, and potential impacts arising from the fellowship.

**Figure:**
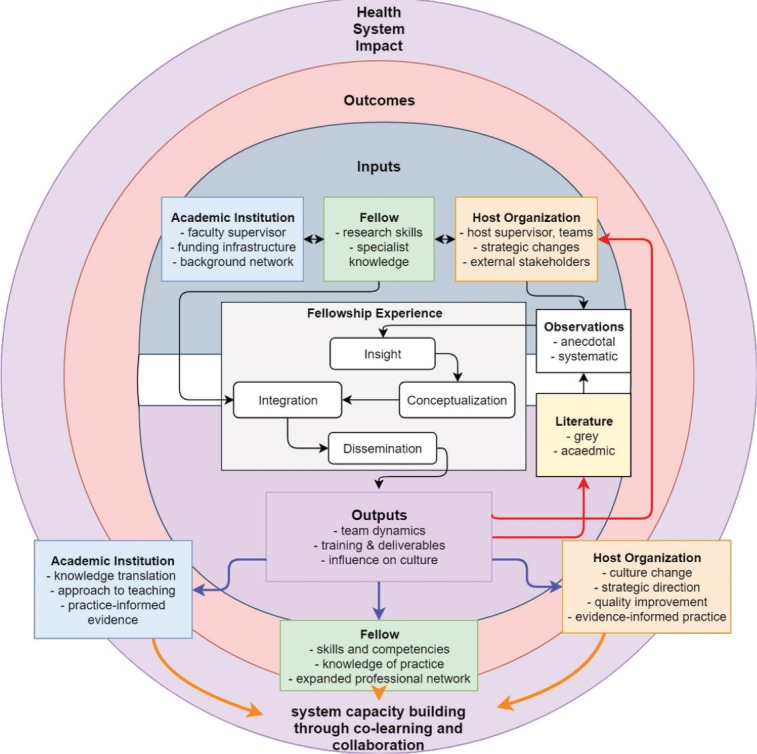
Framework for Understanding the HSI Fellow as an Embedded Researcher. Abbreviation: HSI, Health System Impact.

### Inputs

Inputs (Figure) refer to the fellow’s background and training prior to entering the fellowship, as well as the environments (academic and host organizations) informing their fellowship work. In considering the inputs, we reflected on how we would characterize ourselves with respect to both environments, and how our backgrounds have prepared us (or not) for our current roles. For example, several of the authors are current or formerly licensed health professionals, or have worked in health service, administration, or policy roles. Past experiences within the academic environment (as community-based health researchers, research coordinators or associates, post-doctoral fellows, lecturers) also enables critical reflection of social-political relations along with an understanding of context-relevant pedagogical processes. These practice-based and person/client-focused experiences provide an immediate foundation for the “culture” and organizational behaviour that exists within health service and policy-oriented environments. Moreover, these prior experiences support the necessary networking, trust, and relationship-building processes as enablers for effective multi-sectoral collaboration^7^ to identify and address critical health system challenges through IKT. While each host environment is unique in its current use of PhDs and academic-oriented personnel, the fellowship is a deliberate attempt to bridge the knowledge-to-practice gap and contribute to evidence-informed policy planning and the delivery of care and services.

### Fellowship Environments

Within the practice environment, the fellows’ past experiences, the observations and reflections made by them, and their interactions from proximity with knowledge users^16^ are used in combination with the extant literature (academic and grey literature) and tacit knowledge to inform the work. Rarely are traditional academic outputs the sole impetus for transformative HSI.^3^ Rather, the role of the HSI fellow is as the ‘central agent’ who navigates the system in which they are immersed and becomes a conduit for system-level change by interacting with the knowledge users within that system, conceptualizing that knowledge, integrating those schemas into the whole of their past training and experience, and acting as a facilitator to promote the use of evidence, as well as co-producing knowledge that meets the setting’s decision-making needs. Academic supervisors (either in a new relationship or with existing research appointments at host organizations) play a key role along with the fellow to support IKT within the organization by co-producing research, or enabling ways for the fellow to share quality improvement initiatives as recognizable academic outputs.

### Outputs

It would seem counter-intuitive to the goals of a ‘learning health system’ that peer-reviewed literature is not the standard by which decisions are made. Most important is the intersection or co-production of outputs that are created as the host organization and the HSI fellow (with support from their academic supervisor) operate within this mutual learning space with best available evidence and practices at that moment. Thus, in our model, the main outputs are dispensed as grey literature (within the organization), colleagues, and through consultation services to knowledge users in the organization. The first two domains also feed into the work of the knowledge user. This creates contextualized knowledge that addresses the priorities and needs of the host organizations to support innovations and their implementation, to facilitate organizational and practice changes and quality improvement initiatives, and inform IKT work. The outputs of this fellowship may include academic publications; however, outputs also have the potential to promote and validate practical wisdom, to develop organizational capacity and value for research,^7^ and inform change within the host organization, all while supporting professional growth of the fellow while instilling a “decision-relevant” perspective.^12^ Further, as a co-learning opportunity for the host organization and their academic supervisor, these outputs reintegrate into the experience in a continuous manner through the fellow’s systematic and anecdotal observations (red arrows, Figure).

### Outcomes

We summarize the outcomes of this fellowship based on the framework presented as follows: individual, the environments (academic and host organizations), and the broader health system as a learning health system. The outputs are transformed into outcomes (blue arrows, followed by the orange arrows, Figure) over time as change occurs within each setting.

#### Individual

Practical experience in a real world context supports applied and integrated research, and relevant post-doctoral training. This program positions fellows alongside key decision-makers within the health system (senior leaders, managers, and clinicians) to support “decision-relevant” perspectives^12^; tailoring professional competencies according to the current realities of employment outside the academy, while also maintaining close relationships with universities and research centres. For the fellows who are also health professionals, these experiences can also enhance frontline practice by facilitating a ‘systems-thinking’ perspective regarding health service and delivery. Another potential outcome is for fellows to become key contributors to the decision-making processes within host organizations by promoting knowledge of best practices. Altogether, these opportunities allow the fellow to learn how academic research is applied in ‘real-world’ contexts, which may better align approaches to research intended to impact policy and practice and its implementation.^12^

Further, the opportunity to interact with other fellows in the cohort was an especially valuable mechanism for peer support. Despite heterogeneous experiences, a common exposure as an embedded health researcher allowed for rich exchange about challenges and potential solutions that could be adapted to each fellow’s context (eg, a voluntary group of fellows working on scholarly activities together is but one outcome of this deliberate interaction). Expansion of peer networking opportunities among the cohort could potentially magnify any impacts made across host and academic organizations.

#### Environment

For the academic organization, the fellowship: (*a*) consolidates its links with health system organizations in the context of concrete projects supporting transformation of health services and policy; (*b*) enables a process to sustain the experiential learning for HSI fellows, which in turn increases their chances of career success and provides employers with a skilled workforce; and (*c*) highlights opportunities for innovation in research and development – specifically through IKT.

For fellows who also maintained academic teaching positions, integration between the academic and host organizations manifested in unexpected ways. For example, the fellowship experience informed changes in course content and pedagogical approaches, through the development of new research-policy collaborations, and by the fellows’ participation on multi-sectoral boards and committees.

For the host organization, the fellowship: (*a*) enables the organization to position itself as a learning organization to meet the challenge of innovation, knowledge translation, and transformation in a complex health network; (*b*) improves its service offering in the context of change management and the development of health innovation projects; and (*c*) builds capacity for co-producing evidence.

#### Health System

For the health system, the fellowship: (*a*) provides access to highly qualified personnel who exercise leadership to improve the performance of the Canadian health system; (*b*) allows researchers to work with knowledge users in health services and policy research outside of a traditional academic setting; and (*c*) promotes new partnerships between the host and academic organizations, including cross-sectoral collaborations beyond the health system.

### Impact and Future Directions

Impact is routinely represented as a long-term outcome not easily demonstrable during the life cycle of a project. This fellowship prioritizes impact and accelerates the process by positioning the fellow within an organizational program of work which precedes the fellow, and exceeds a single project or intervention. This creates a unique opportunity for fellows, host, and academic to prioritize, experience, evaluate, and even shape impact together. For example, mutual learning about intersectoral, participative, and interdisciplinary research occurs while operating in bi-directional knowledge transfer between academic and scientific spaces, and in decision-making, practice and communities. Overall, this set up recognizes that impact is a collective endeavour occurring within complex adaptive systems.

We recognize that there are implications for fellows who want to pursue a career that bridges both the health system and academic environments. It is unresolved how the training fellowship will impact future academic success. We suggest that the HSI fellowship facilitates reflection on how the academy sees value in contributions outside of traditional academic outputs. An important direction for this work should explore this question for fellows who continue work within the academic space pertaining to how and what knowledge is valued. Future research should also explore the challenges that arise through embedded researcher initiatives, and provide a more in-depth examination into the program’s contributions and impact from the perspective of fellows, academic, and host supervisors.

## Conclusion

The HSI Fellowship is helping to “drive change” and modernize the health system. A fellowship of this kind presents many opportunities. We recognize the HSI Fellowship as enabling us to sit on the precipice of health system transformation through our important role as trainees and collaborators. This inaugural fellowship is a tangible initiative that can meet the challenge of innovation and adaptation within a dynamic and complex health system landscape.

## Acknowledgements

Financial support of this work was provided to all authors through the Canadian Institutes of Health Research’s (CIHR) Health System Impact Fellowship. This program is led by CIHR’s Institute of Health Services and Policy Research (CIHR-IHSPR), in partnership with the CIHR Institutes of Aging (CIHR-IA), Aboriginal Peoples’ Health (CIHR-IAPH), Cancer Research (CIHR-ICR), Circulatory and Respiratory Health (CIHR-ICRH), Infection and Immunity (CIHR-III), Nutrition, Metabolism and Diabetes (CIHR-INMD), Population and Public Health (CIHR-IPPH), and the Science, Knowledge Translation and Ethics (SKTE) Branch and external partners, including the Fonds de recherche du Québec –Santé (FRQS) and Mitacs. JL’s fellowship is co-funded by CIHR-IHSPR and Mitacs.

## Ethical issues

Not applicable.

## Competing interests

Authors declare that they have no competing interests.

## Authors’ contributions

All authors provided intellectual contribution to this article. SMS and JL drafted the article. All authors edited, provided written contributions, and reviewed the final version. This article is a condensed summary of a panel presentation held at the May 2018 Canadian Association for Health Services and Policy Research annual conference in Montréal, QC, Canada.

## Authors’ affiliations

^1^Healthy Populations Institute, Dalhousie University, Halifax, NS, Canada. ^2^Nova Scotia Health Authority, Halifax, NS, Canada. ^3^McGill University, Montreal, QC, Canada. ^4^Centre for Innovation in Autism and Intellectual Disabilities, Montreal, QC, Canada. ^5^Mount Saint Vincent University, Halifax, NS, Canada. ^6^Continuing-Care Research, Nova Scotia Health Authority, Halifax, NS, Canada. ^7^Institute of Health Policy and Management, University of Toronto, Toronto, ON, Canada. ^8^Sunnybrook Health Sciences Centre, Toronto, ON, Canada. ^9^DeGroote School of Business, McMaster University, Hamilton, ON, Canada. ^10^Nova Scotia Health Research Foundation, Halifax, NS, Canada. ^11^Institut National D’excellence en Santé et en Services Sociaux (INESSS), Québec City, QC, Canada. ^12^Centre Intégré en Santé et Services Sociaux de Chaudière-Appalaches, Ste-Marie, QC, Canada. ^13^Département de Médecine Familiale et Médecine D’urgence, Université Laval, Quebec City, QC, Canada. ^14^School of Public Health, University of Alberta, Edmonton, AB, Canada. ^15^Primary and Community Health, Health Service Delivery, Alberta Health, Edmonton, AB, Canada. ^16^Department of Human Kinetics, University of Ottawa, Ottawa, ON, Canada. ^17^University Health and Social Services Centre (IUHSSC) of Capitale-Nationale (CN), Faculty of Nursing Sciences, Université Laval, Québec City, QC, Canada. ^18^School of Public Health and Health Systems, University of Waterloo, Waterloo, ON, Canada. ^19^SE Health, Markham, ON, Canada. ^20^Department of Epidemiology Epidemiology, Biostatistics and Occupational Health, McGill University, Montréal, QC, Canada. ^21^Institut National de Santé Publique du Québec (INSPQ), Montréal, QC, Canada. ^22^Saskatchewan Health Authority, Saskatoon, SK, Canada. ^23^University of Saskatchewan, Saskatoon, SK, Canada.
